# The Influence of Serum Iron Levels on Depression, Anxiety, Fatigue, Neuropathic Pain and MR Disease Activity in Patients with Multiple Sclerosis

**DOI:** 10.3390/neurosci7040086

**Published:** 2026-07-27

**Authors:** Simonida Delic, Svetlana Miletic Drakulic, Snezana Lazarevic, Milos Stepovic, Nikoleta Janicijevic, Maja Vulovic, Aleksandra Mitrovic Zivanovic, Danica Igrutinovic, Melanija Tepavcevic, Milica Dimitrijevic, Katarina Manojlovic, Bojana Markovic, Nebojsa Igrutinovic, Vladimir Markovic, Ana Azanjac Arsic

**Affiliations:** 1 Department of Anatomy, Faculty of Medical Sciences Kragujevac, University of Kragujevac, 34000 Kragujevac, Serbia; simonida.delic@fmn.kg.ac.rs (S.D.); stepovicmilos@yahoo.com (M.S.); maja@fmn.kg.ac.rs (M.V.); melanijatepavcevic@yahoo.com (M.T.); milicadimitrijevic479@gmail.com (M.D.); 2 Department of Neurology, Faculty of Medical Sciences Kragujevac, University of Kragujevac, 34000 Kragujevac, Serbia; mileticdrakulic@fmn.kg.ac.rs (S.M.D.); ana.azanjac@fmn.kg.ac.rs (A.A.A.); 3 Department of Hygiene and Ecology, Faculty of Medical Sciences Kragujevac, University of Kragujevac, 34000 Kragujevac, Serbia; nikoleta.janicijevic@gmail.com; 4 Department of Gynecology and Obstetrics, University Clinical Centre Kragujevac, 34000 Kragujevac, Serbia; mitrovicaleksandra573@gmail.com; 5 Department of Biochemistry, University Clinical Centre Kragujevac, 34000 Kragujevac, Serbia; danica7pavlovic@gmail.com; 6 Department of Plastic Surgery, University Clinical Centre Kragujevac, 34000 Kragujevac, Serbia; 7 Department of Physical Medicine and Rehabilitation, University Clinical Centre Kragujevac, 34000 Kragujevac, Serbia; manojlovickatarina112@gmail.com; 8 Department of Pediatrics, University Clinical Centre Kragujevac, 34000 Kragujevac, Serbia; bojana.kovacevic96@gmail.com; 9 Department of Internal Medicine, University Clinical Centre Kragujevac, 34000 Kragujevac, Serbia; shone31094@gmail.com; 10 Department of Microbiology and Immunology and Fundamentals of Oncology, Faculty of Medical Sciences Kragujevac, University of Kragujevac, 34000 Kragujevac, Serbia; vladimirmarkovic.vlad@gmail.com; 11 Department of Microbiology and Immunology, University Clinical Centre Kragujevac, 34000 Kragujevac, Serbia

**Keywords:** iron, multiple sclerosis, disease activity

## Abstract

Background: Multiple sclerosis (MS) is a chronic inflammatory, autoimmune, and neurodegenerative disease of the central nervous system. Our study aimed to examine the relationship between serum iron levels and the following clinical parameters: depression, anxiety, fatigue, neuropathic pain, and disease activity on magnetic resonance imaging in patients with relapsing-remitting multiple sclerosis (RRMS). Material and methods: This study was designed as a clinical observational, cross-sectional study. This study was conducted at the Clinic of Neurology of the University Clinical Center Kragujevac, from 2022 to 2024, according to the ethical code of the Declaration of Helsinki. Demographic and clinical data on patients were obtained from the medical history. All MS patients were diagnosed according to the McDonald criteria (2017). This study included 65 patients in the experimental group and 11 healthy individuals in the control group, with an average age of 39.68 years. Serum iron levels were biochemically analyzed from venous blood. The following standardized tests were used to collect data on depression, anxiety, fatigue, and neuropathic pain: Beck Depression Inventory (BDI), Hamilton Anxiety Rating Scale (HAM-A), Modified Fatigue Impact Scale (MFIS), PainDETECT Questionnaire (PD-Q), and Visual Analogue Scale (VAS). Results: Serum iron level significantly correlates with disease activity on magnetic resonance imaging in patients with multiple sclerosis. Serum iron showed a moderate positive correlation with hemoglobin, hematocrit, total and direct bilirubin, and a weak positive correlation with uric acid, while a moderate negative correlation was observed with erythrocyte sedimentation rate. No statistically significant difference was found between serum iron levels and depression, anxiety, fatigue, and severity of neuropathic pain. The ROC curves show the diagnostic potential of the combined parameters in differentiating RRMS from controls, as well as the potential of iron in differentiating active from inactive MS. The presence of disease, hemoglobin and total bilirubin were recorded as significant independent predictors. Conclusion: This study is a descriptive attempt to present the potential role of iron in the radiological activity of the disease. High serum iron level may be associated with radiological disease activity in patients with multiple sclerosis.

## 1. Introduction

Multiple sclerosis is a neuroinflammatory and neurodegenerative disorder of the central nervous system (CNS) and is characterized by lesions in the gray and white matter. The disease impacts the brain, spinal cord, and optic nerves. The primary processes in multiple sclerosis are neuroinflammation and neurodegeneration. These two processes coexist from the outset, with neuroinflammation dominant in the relapsing-remitting form, whereas neurodegeneration is predominant in the primary progressive and secondary progressive forms [1]. The underlying process leading to progression in RRMS is latent, smoldering activity. Irreversible, progressive disability accumulation can happen at any stage of RRMS through mechanisms such as relapse-associated worsening (RAW) and progression independent of relapse activity (PIRA). The mechanisms behind PIRA are believed to involve chronic inflammation and neurodegeneration that begin very early in the disease course [2]. The 2013 Lublin classification divides the phenotypes into RRMS, secondary progressive multiple sclerosis (SPMS), and primary progressive multiple sclerosis (PPMS). This older classification is gradually being revised, and today it is considered that RRMS and SPMS are simply two ends of the same spectrum [3]. Based on clinical and radiological activity, the disease can be classified as inactive or active. Clinical disease activity refers to the number of relapses in one year and over two years, measured by the annual relapse rate (ARR). ARR is used in clinical studies as an outcome measure for multiple sclerosis and helps determine the required sample size. Radiological disease activity involves the presence of T1 lesions enhanced by gadolinium contrast on T1-weighted sequences, or an increase in the number or volume of new T2 lesions compared to the previous MRI performed a year earlier [4].

In our organism, iron is found in hemoglobin, myoglobin, digestive enzymes and stored as ferritin. In the brain, iron, as an enzyme cofactor, participates in the process of neurotransmission and myelin synthesis. Also, iron plays a significant role in the remyelination process [5]. Oligodendrocytes are myelin-forming cells that contain large reserves of iron, while astrocyte extensions are actually part of the blood–brain barrier and exchange substances through them. Damage to both cell types caused by oxidative stress would potentially contribute to iron accumulation and disruption of the blood–brain barrier with increased permeability, which would lead to the entry of new immune system cells, excessive and constant activation of the pro-inflammatory phenotype of microglia with increased neuroinflammation [6]. Studies have shown that in active lesions of multiple sclerosis, the breakdown of myelin and subsequent phagocytosis of its remains occur. During myelin breakdown, iron is released into the extracellular space, enhancing oxidative stress in multiple sclerosis lesions. The iron is then taken up by macrophages and microglia. After their degeneration, iron is again released into the extracellular space, triggering a new wave of oxidative stress [7]. Studies suggest that high iron levels induce inflammation and act as a catalyst for the production of reactive oxygen species that play a significant role in the pathogenesis of many neurodegenerative diseases, including multiple sclerosis. Pathophysiologically, it is based on the activation of microglia, which generates an increased concentration of free radicals in the presence of iron ions originating from damaged cells. There are slowly expanding lesions surrounded by phagocytes, a ring of microglial cells that internalize free iron [3]. Oxidative stress is a consequence of mitochondrial dysfunction, where free iron can stimulate increased production of free radicals in the respiratory chain. Mitochondrial dysfunction exists in both glial cells in the CNS and immune cells, where energy production is actually redirected from oxidative phosphorylation, which is a highly efficient process, to the less efficient anaerobic glycolysis, which releases lactate. Lactate further contributes to the maintenance of neuroinflammation and the multiplication of oxidative stress to the point where apoptosis mechanisms are activated, which begins the death of neurons and glial cells, which manifests as neurodegeneration [6]. Also, serum iron level correlates with markers of disease progression [8,9]. In patients with multiple sclerosis, there is evidence of iron deposition in the brain based on magnetic resonance imaging [10]. The early appearance of iron rim lesions is associated with active demyelination, while over time these lesions decrease and disappear or transform into lesions without an iron rim [11,12]. According to data from the literature, the relationship between parameters of iron metabolism and fatigue and depression in patients with stroke [13], Parkinson’s disease [14], and fibromyalgia [15] has been investigated. Knyzinska et al. investigated the relationship between fatigue and depression and parameters of iron metabolism in patients with multiple sclerosis [16]. Zierfuss et al. reviewed iron metabolism, the nervous system cells involved in its metabolism and turnover, and iron chelator and antioxidant therapy, which are being investigated but have not yet yielded significant effects [17]. Emamnejad et al. discussed the role of iron in the process of ferroptosis, cell death induced by iron excess, as well as ferritinophagy, a process in which redox-active iron is released from ferritin stores. Previous studies on experimental models of experimental autoimmune encephalomyelitis (EAE) have shown beneficial effects of ferroptosis inhibitors [18]. Iron storage in cells is most often in the form of ferritin. Riedl et al. noticed that there is a disturbance in the level of ferritin under inflammatory conditions on tissue samples obtained at autopsy and by immunohistochemistry [19]. A divalent metal transporter transports it within the cell; export from the cell occurs via ferroportin, and transport via transferrin. Hepcidin is a molecule that regulates the passage of iron across the blood–brain barrier. During neuroinflammation, increased expression of these molecules leads to iron retention in cells [17,20,21]. Erythrocytes in MS are fragile, and hemoglobin is released in an increased manner by hemolysis from erythrocytes and can cross the blood–brain barrier. The passage of the blood–brain barrier occurs via transferrin receptors. Free hemoglobin in brain tissue leads to neurodegeneration and brain shrinkage [22]. A possible mechanism that would link peripheral iron to that deposited in the CNS would be the spillover of excess extracellular iron, where iron transporters would return free iron across the blood–brain barrier into the circulation, but this has not yet been proven. The cause could potentially be increased permeability of the blood–brain barrier or increased expression of iron transporters induced by neuroinflammation and oxidative stress.

Iron accumulation increases with age, while in MS, the level of iron increases rapidly in the basal ganglia and decreases in the white matter of normal appearance [23].

The relationship between iron levels and radiological activity of the disease on MRI has not been investigated so far, but there are studies that compare iron levels between patients with MS and healthy controls. A small number of studies have been conducted on the association of iron with clinical symptoms such as neuropathic pain, anxiety, depression, and fatigue. Most of the studies are from other geographical areas, and it is known that there are geographical differences in iron metabolism, as well as genetic variants. This would be the first study in the region of Southeastern Europe, the first study in Serbia that links these parameters. Given the shortcomings in previous research, our study aimed to examine the association of serum iron levels with radiological activity, level of depression, level of fatigue, level of anxiety, the presence of neuropathic pain, and pain severity in patients with MS.

## 2. Materials and Methods

### 2.1. Study Design

This study was conducted as a clinical observational, cross-sectional study.

### 2.2. Participants

This study was conducted at the Clinic of Neurology, University Clinical Center Kragujevac, from 2022 to 2024, according to the ethical code of the Declaration of Helsinki. Demographic and clinical data on patients were obtained from their medical history. All patients were included in this study voluntarily. The data obtained were anonymized. The Ethics Committee of the University Clinical Center Kragujevac approved this study. The study participants were patients with RRMS and healthy, non-smoking individuals. All patients with multiple sclerosis were diagnosed according to the McDonald criteria (2017) [24].

The control group consisted of healthy, non-smoking individuals over 18 years of age, matched by sex and age with the patients in the study group. All study participants signed an informed consent form, in which they were informed, both in writing and orally, about the nature of the research, the absolute confidentiality of the data obtained, and its use exclusively for scientific purposes.

### 2.3. Blood Sampling and Questionnaires

On the same day, 5 ml of blood was taken from the patients from a peripheral vein in a tube in the morning on an empty stomach for iron level analysis, and questionnaires were filled out to assess the level of depression, fatigue, anxiety, the presence of neuropathic pain, and pain intensity, as well as MRI scans. The manufacturer of chemicals and reagents is Sigma-Aldrich Company, Darmstadt, Germany.

#### Inclusion and Exclusion Criteria

Inclusion criteria were patients diagnosed with RRMS according to the McDonald criteria (2017), patients older than 18 years of age, sex (female/male), and who signed informed consent. Included patients were neurologically stable without relapse for 3 months before inclusion in this study. Patients were treatment-naive, because at that time in Serbia, medium-efficacy and high-efficacy DMT was not available in clinical centers.

Exclusion criteria were patients with PPMS and SPMS according to the McDonald criteria (2017), with RRMS who received disease-modifying therapy or corticosteroid therapy within 3 months before inclusion in this study, patients who used vitamins, antioxidants, drugs that may interfere with iron metabolism, anti-inflammatory or hormonal therapy within 3 months before inclusion in this study, patients with other neurological diseases that may interfere with the diagnosis of multiple sclerosis, as well as a history of neuromyelitis optica, other immunological diseases, the presence of injuries and tumors of the central nervous system, a history of malignancy of any organ system and patients who were not smokers in the last year. Patients with uncontrolled, clinically significant systemic diseases such as uncontrolled diabetes mellitus, cardiovascular diseases, renal, hepatic, pulmonary, gastrointestinal, hematological, and psychiatric diseases were also excluded.

This study included 65 patients in the experimental group and 11 patients in the -control group, with an average age of 39.68 years.

### 2.4. Variables Measured in This Study

The independent variables were demographic and clinical parameters that were monitored: age, sex, presence of disease, age of diagnosis (years of age when the diagnosis was established), time from the onset of neurological symptoms to admission to the clinic, duration of the disease, clinical activity of the disease, radiological activity of the disease on magnetic resonance imaging of the head, findings of isoelectric focusing of cerebrospinal fluid and serum, severity of the clinical picture, measured byKurtzke’s Extended Disability Status Scale (EDSS) laboratory parameters obtained from the medical history (sedimentation rate, leukocyte count, erythrocyte count, hemoglobin, hematocrit, iron, C-reactive protein (CRP), fibrinogen, bilirubin, vitamin D, uric acid), as well as level of depression, measured by the Beck Depression Inventory (BDI), a scale for assessing depression; level of anxiety, measured by the Hamilton Anxiety Rating Scale (HAM-A); level of fatigue, measured by the Modified Fatigue Impact Scale (MFIS); the presence of neuropathic pain, measured by the PainDETECT Questionnaire (PD-Q); and pain intensity, measured by the Visual Analogue Scale (VAS).

The age of the subjects was grouped into three categories (≤30, 31–60, and ≥61 years), and similar age divisions have been made in previous studies [25,26]. We divided the subjects according to the duration of the disease into groups 0–10 years, 11–20 years, and 21–40 years. We made this division according to the model of the previous division of the duration of the disease [27]. We also divided the subjects by disease onset before 55 years and after 55 years, referring to the cutoff for late-onset MS [28].

Oxidative stress parameters were determined from blood samples. We measured the values of pro-oxidative markers: lipid peroxidation index, thiobarbituric acid (TBARS), nitrites (NO_2_^−^), superoxide anion radical (O_2_^−^), hydrogen peroxide (H_2_O_2_), and the activities of the antioxidant defense system enzymes: catalase (CAT), superoxide dismutase (SOD), and reduced glutathione (GSH). All of the above parameters were measured spectrophotometrically. 

#### 2.4.1. Determination of Lipid Peroxidation Index

The test involves the reaction of the lipid peroxidation product, malondialdehyde (MDA), with thiobarbituric acid (TBA), to form MDA-TBA2 complexes called TBARS. In this procedure, distilled water was used as a blank. 0.8 mL of the sample and 0.4 mL of trichloroacetic acid were mixed. The supernatant was kept on ice for 15 min, and centrifuged at 6000× *g*, and stored. After that, 1% thiobarbituric acid in 0.05 NaOH was separated from the supernatant at 100 °C for 15 min. The measurement was carried out at a wavelength of 530 nm using a spectrophotometer [29].

#### 2.4.2. Determination of Nitrite Concentration

In this reaction, a distilled water solution was used as a blank. The NO_2_^−^ level was determined using the Griess reagent-based NO production. First, 0.1 mL of 3 N perchloric acid, 0.4 mL of 20 mM ethylenediaminetetraacetic acid, and 0.2 mL of the sample were kept on ice for 15 min, then centrifuged for 16 min at 6000× *g* rpm. The supernatant was poured off, and 220 μL of K_2_CO_3_ was added. NO_2_ was measured at a wavelength of 550 nm spectrophotometrically [30].

#### 2.4.3. Determination of Hydrogen Peroxide

In this reaction, distilled water was used as a blank. Hydrogen peroxide (H_2_O_2_) determination was performed in a peroxidase-catalyzed reaction (HRPO) where phenol is oxidized by hydrogen peroxide. A sample of 800 μL of freshly prepared red phenol solution was mixed with 200 μL of plasma, and then 10 μL (1:20) HRPO was added. The H_2_O_2_ level was measured at a wavelength of 610 mm spectrophotometrically [31].

#### 2.4.4. Determination of Superoxide Anion Radicals

The principle of determination of superoxide radicals (O_2_) is the reaction of nitro blue tetrazolium in TRIS buffer with plasma samples. Distilled water was used as a blank. The superoxide anion was measured at a wavelength of 530 nm spectrophotometrically [32].

#### 2.4.5. Determination of Catalase

The Beutler method was used to measure catalase, combining 50 L of CAT buffer, 100 L of sample, and 1 mL of 10 mM H_2_O_2_. The rate of decomposition of hydrogen peroxide in the presence of catalase was monitored and recorded at a wavelength of 360 nm. Distilled water served as a blank, and the amount of CAT was expressed as U/g hemoglobin 103 [33].

#### 2.4.6. Determination of Superoxide Dismutase

The epinephrine technique for determining SOD activity is a method of monitoring the decrease in the rate of adrenaline autooxidation in an alkaline medium, which is dependent on O_2_^−^. First, 100 L of the sample was mixed with 1 mL of carbonate buffer, and then 100 L of epinephrine was added to the test tube. SOD activity was measured at a wavelength of 470 nm spectrophotometrically and expressed as U/g hemoglobin 103. Because SOD removes O_2_^−^, inhibition of the reaction occurs [34].

#### 2.4.7. Determination of Reduced Glutathione

In this reaction, distilled water was used as a blank probe, and measurements were performed at a wavelength of 420 nm spectrophotometrically. To prepare the GSH extract, 0.1 mL of 0.1% EDTA, 400 mL of plasma, and 750 mL of precipitation solution (1.67 g metaphosphoric acid, 0.2 g EDTA, 30 g NaCl, and 100 mL of distilled water) were mixed. It was kept on ice for 15 min, and mixed in a vortex device; the mixture was centrifuged at 4000× *g* for 10 min. Based on the oxidation of GSH with 5,5-dithiobis-6,2-nitrobenzoic acid, the amount of reduced glutathione (GSH) was measured. The concentration is expressed as nanomoles per milliliter of red blood cells [35].

#### 2.4.8. Extended Disability Status Scale—EDSS Score in the Assessment of Multiple Sclerosis

Kurtzke’s Extended Disability Status Scale-EDSS from 1983. monitors functional systems: visual (optical) functions, brainstem functions, pyramidal functions, cerebellar functions, sensory functions, bowel and bladder functions, cerebral functions, and ambulatory function [36]. We classified the EDSS score into mild (0–3.5), moderate (4–6), and severe (≥6.5) based on previous studies [37]. Somilo et al. conducted a study with 105 patients with RRMS in two groups according to the EDSS score: if the EDSS score was less than 3.5—mild disability, if it was from 3.5 to 6.5—moderate disability [38]. A systematic review of the literature considered the cut-off values, validity, and reliability of the EDSS scale [39].

#### 2.4.9. Beck Depression Inventory for the Assessment of Multiple Sclerosis

The Beck Depression Inventory is a self-report scale for screening for depression and measures the severity of depression. The items scored are emotional, cognitive, and somatic symptoms of depression. It is intended for adults and adolescents aged 13 years and older and is a revision of the original BDI from 1961.Concurrent validation studies of the BDI with other self-rating scales for depression place it in the classification of moderate to strong validation. Each of the 21 items in the BDI-II is followed by four statements that represent increasing levels of distress from 0 to 3. The subject is instructed to circle the number next to the statement that best represents how the subject “has felt during the past 2 weeks, including today.” The scores for each item are summed, and the total score ranges from 0 to 63. The cutoff scores and ranks are as follows: 0–13 minimal, 14–19 mild depression, 20–28 moderate depression, 29–63 severe depression [40,41].

#### 2.4.10. Hamilton Anxiety Rating Scale in the Evaluation of Multiple Sclerosis

The HAM-A was one of the first rating scales developed to measure the severity of anxiety symptoms, and is now widely used in clinical and research settings. The 14-item version is the most commonly used outcome measure in clinical trials of anxiety treatment. It measures both psychological anxiety (mental distress and psychological distress) and somatic anxiety (physical symptoms related to anxiety). Each item is scored on a scale from 0 (none) to 4 (severe), with less than 17 indicating mild severity, 18–24 indicating mild to moderate severity, and 25–30 indicating moderate to severe. The maximum score is 56. Although depressive symptoms can overlap with anxiety symptoms, this test is reliable, valid, and sensitive to change [42,43].

#### 2.4.11. Fatigue Impact Scale (MFIS) in the Assessment of Multiple Sclerosis

The Modified Fatigue Impact Scale (MFIS) is an instrument for assessing the impact of fatigue on the quality of life in patients with MS. It is a modified version of the original 40-item Fatigue Impact Scale (FIS). The MFIS evolved from the FIS during the development of a clinical inventory for assessing the overall quality of life of people with MS, the Multiple Sclerosis Quality of Life Inventory (MSQLI). The MFIS has 21 items divided into three domains: physical, cognitive, and psychosocial. The items are rated on a scale from 0 to 4, where 0 indicates “no problem” and 4 indicates “extreme problem”. The maximum possible score is 84, with higher scores indicating a greater impact on quality of life. This instrument has been validated in the MS patient population and is recommended in clinical practice and research [44]. A score of 38 was determined as the cut-off value between patients, separating patients without fatigue from patients with fatigue [45,46]. One study examined fatigue thresholds by gender, age group, and years of education [47].

#### 2.4.12. PainDETECT Questionnaire in the Evaluation of Multiple Sclerosis

The PainDETECT Questionnaire is a screening method for assessing the likelihood of having neuropathic pain. It was developed in a study investigating chronic low back pain [48]. It contains questions about pain in the last four weeks, the course of the pain, the main area of pain, burning, tingling, pain with light touch and pressure, heat and cold, numbness, and sensations like electric shocks. The patient grades the answers from none, barely noticeable, mild, moderate, severe, to very severe. The total score is 35 points. The patient receives additional points if they have attacks of pain with and without the appearance of pain between and with the spread of pain (3 maximum). A negative score is taken if the patient has 12 or less points with a low probability of the presence of a neuropathic pain component (<15%), an unclear score is for scores 13–18, but a neuropathic pain component may be present, and a positive score is for scores 19–38 with a high probability of the presence of a neuropathic pain component (>90%) [49]. Another study concluded that PD-Q reliably and validly discriminates pain severity in patients with neuropathic pain. The advantage of this questionnaire is that it does not require a clinical examination [50].

#### 2.4.13. Visual Analogue Scale for Pain Assessment (VAS)

The Visual Analogue Scale (VAS) is a one-dimensional instrument for assessing the intensity of pain. On a straight line of 100 mm in length, the subject should mark a point that represents the intensity of pain at the time of the examination. Pain is classified as mild (1–30 mm), moderate (31–69 mm), and severe (≥70 mm). The advantages of this scale are that it is quick to use and easy to understand for most patients, it avoids descriptive terms, and it is possible to compare and repeat measurements. It is not suitable for patients in the postoperative period because it requires cognitive engagement and visual impairment. If the patient experiences the most severe pain ever and then experiences an even greater intensity of pain, this change cannot be recorded, which is a disadvantage [51].

#### 2.4.14. Study Power and Sample Size

The sample size calculation was performed using G*Power software version 3.1.9.4. The primary objective of this study was to compare biomarkers of iron metabolism between patients with relapsing-remitting multiple sclerosis (RRMS) and healthy controls. As no previously published study with an identical design, study population, and set of investigated biomarkers was available, the expected effect size was estimated using data from a study evaluating iron metabolism parameters in patients with multiple sclerosis and healthy individuals [52]. The anticipated between-group difference and variability of measurements were determined based on the reported mean values and standard deviations of relevant biomarkers, while also taking into account biologically and clinically plausible assumptions regarding the expected magnitude of differences in our study population. An independent-samples t-test was used as the reference statistical test for sample size estimation. The significance level (α) was set at 0.05 and statistical power at 80%. Based on the anticipated effect size derived from the available literature, the minimum required sample size was estimated to be 48 participants. Because deviations from normal distribution were considered possible and nonparametric statistical methods might be required, the target sample size was conservatively increased by approximately 15%, resulting in a recommended minimum sample of 56 participants. The final study included 65 patients with RRMS and 11 healthy controls (total n = 76), exceeding the calculated minimum sample size and thereby providing adequate statistical power for the planned analyses. The sample size estimation was performed to minimize random error and reduce the risk of both type I (false-positive) and type II (false-negative) findings.

#### 2.4.15. Statistical Data Processing

The variables examined in this study were processed in the commercially available standard software package SPSS 26.0 (IBM, Chicago, IL, USA).

Three types of data were analyzed: qualitative data (such as gender, disease form, clinical activity of the disease, radiological activity of the disease of patients), quantitative discrete data (age, time from the onset of neurological symptoms to admission to the clinic, duration of the disease, and the severity of the clinical picture—EDSS) and continuous quantitative data (sedimentation rate, leukocyte count, erythrocyte count, hemoglobin, hematocrit, iron, C-reactive protein, fibrinogen, bilirubin, and vitamin D). To assess the normality of the results, we used the Kolmogorov-Smirnov test. If the data followed a normal distribution, we used parametric tests (an independent-samples t-test and a one-way ANOVA). If the data did not follow a normal distribution, we used nonparametric techniques (the Mann–Whitney U test and the Kruskal–Wallis test). To determine the relationship between variables, whether it was positive or negative, we used correlation. We used Spearman’s coefficient because serum iron did not follow a normal distribution. To assess the influence of a variable on another numerical variable, we used single linear regression. The significance level was set at *p* < 0.05. The confidence interval taken into account was set at 95%.

We presented the data using frequencies, measures of central tendency (mean and median), measures of variability (variance, standard deviation, 25th–75th quartile (interquartile range), 95% confidence interval, standard error), in tables and graphs. Data that follow a normal distribution are presented as the mean and standard deviation. Data that do not follow a normal distribution are presented as the median and 25th–75th percentile. The ROC curve is presented as a graph, and numerically expressed as the area under the curve, 95% confidence interval, standard error, and *p* value.

## 3. Results

### 3.1. Demographic and Clinical Characteristics

This study included 26 men (34.2%) and 50 women (65.8%). Of these, 23 men (35.4%) had RRMS, and 42 women (64.6%) had RRMS. Of the total number of respondents, 65 had RRMS (85.5%), while the control group included 11 healthy respondents (14.5%).

Data on radiological disease activity were available for 51 subjects: 39 (76.5%) had no new lesions, 11 (21.6%) had up to three new lesions, and one (2.0%) had more than three new lesions on follow-up MRI. Clinical disease activity was assessed in 54 patients, of whom 23 (42.6%) had inactive disease and 31 (57.4%) had active disease. Oligoclonal bands were assessed in 57 patients and were present in 48 (84.2%) and absent in nine (15.8%). Neuropathic pain was evaluated in 74 subjects and was present in 15 (20.3%), while nociceptive pain was present in 21 (28.4%). Headache was reported in 15 (20.3%) of 74 subjects. Demographic and clinical characteristics of subjects are presented in Table 1 and Table 2.

Comparison of numerical variables between participants with the relapsing-remitting form of the disease and the control group was performed using the Mann–Whitney U test, as the data distribution did not follow normality. The analysis showed no statistically significant differences between the groups regarding age, body weight, height, BMI values, neuropathic pain intensity, fatigue assessed by the MFIS scale, depression assessed by the Beck scale, anxiety assessed by the Hamilton scale, or pain intensity assessed by the VAS scale (*p* > 0.05 for all variables). A statistically significant difference between the observed groups was found only for iron levels (*p* = 0.037), with participants with the relapsing-remitting form of the disease showing a higher mean rank compared to the control group.

### 3.2. Correlation of Iron with Clinical Parameters, Oxidative Stress Parameters, and Questionnaires for Assessing Depression, Anxiety, Fatigue, and Pain

The correlation of iron with clinical parameters, oxidative stress parameters, and questionnaires for assessing depression, anxiety, fatigue, and pain is presented in Table 3. Correlation analysis was performed using Spearman’s rank correlation coefficient due to the non-normal distribution of certain variables. A negative correlation with serum iron was observed for disease duration, isoelectric focusing of cerebrospinal fluid and serum, EDSS score, all oxidative stress parameters except superoxide dismutase, and the results of the PD-Q, MFIS, BDI, HAM-A, and VAS scales. A positive correlation was shown by serum iron with clinical activity, the number of relapses within one year, the number of relapses within two years, disease activity on magnetic resonance imaging, and the enzyme superoxide dismutase. None of these correlations reached statistical significance.

### 3.3. Analysis of Serum Iron Concentration by Groups of Subjects According to Gender, Age, Disease Onset, Disease Duration, Clinical Activity, Disease Activity on MRI, Presence of Oligoclonal Bands, and Headache

Iron levels according to sex, age, disease onset, disease duration, clinical activity, disease activity on MRI, presence of oligoclonal bands, and headache are presented in Table 4, and the post hoc test for MR activity is presented in Table 5. Serum iron did not show a statistically significant difference according to sex, age groups, disease onset, disease duration, clinical activity, presence of oligoclonal bands, or presence of headache. There were minor differences in median values and variability between groups, but these were not statistically significant.

Analysis of other parameters by sex showed statistically significant values for erythrocytes (m = 4.96 ± 0.37, f = 4.39 ± 0.35, *p* < 0.001), hematocrit (m = 0.43 ± 0.03, f = 0.38 ± 0.03, *p* < 0.001), uric acid (m = 308.36 ± 74.42, f = 245.05 ± 78.87, *p* = 0.003), hemoglobin (m = 149.50, f = 130.50, *p* < 0.001), sedimentation rate (m = 5.00, f = 10.00, *p* = 0.003), and depression (m = 4.00, f = 8.00, *p* = 0.025), and anxiety scores (m = 7.00, f = 10.00, *p* = 0.042). Other parameters did not show a statistically significant difference by gender. For parameters that follow a normal distribution, we compared mean values and standard deviations, while for parameters that do not follow a normal distribution, we compared medians. Nominal clinical variables in the chi-square test did not show a significant association with gender.

Comparison of iron levels between groups defined according to MRI activity was initially performed using the Kruskal–Wallis test. After identifying a statistically significant difference among the groups, post hoc analysis using the Mann–Whitney U test was conducted to determine between which groups the differences were present. A statistically significant difference in iron levels was observed between participants without new lesions and those with up to three new MRI lesions (*p* = 0.027), and participants with up to three new lesions showing higher mean ranks for iron levels. No statistically significant differences were found between participants without new lesions and those with more than three new lesions (*p* = 0.113) nor between participants with up to three new lesions and those with more than three new lesions (*p* = 0.206).

Our results did not show a statistically significant association between serum iron levels and levels of depression, anxiety, fatigue, the presence of neuropathic pain, and pain intensity, regardless of whether the analysis was conducted with continuous outcomes or by group. A trend of decreased serum iron with increasing fatigue was observed, but this finding was not statistically significant. Regression models did not reveal a statistically significant effect of depression, anxiety, fatigue, the presence of neuropathic pain, and pain severity on iron levels, nor did they reveal an individual effect of iron levels on the level of depression, anxiety, fatigue, the presence of neuropathic pain, and pain severity.

### 3.4. ROC Curve of Iron as a Biomarker of MS

**Figure 1:**
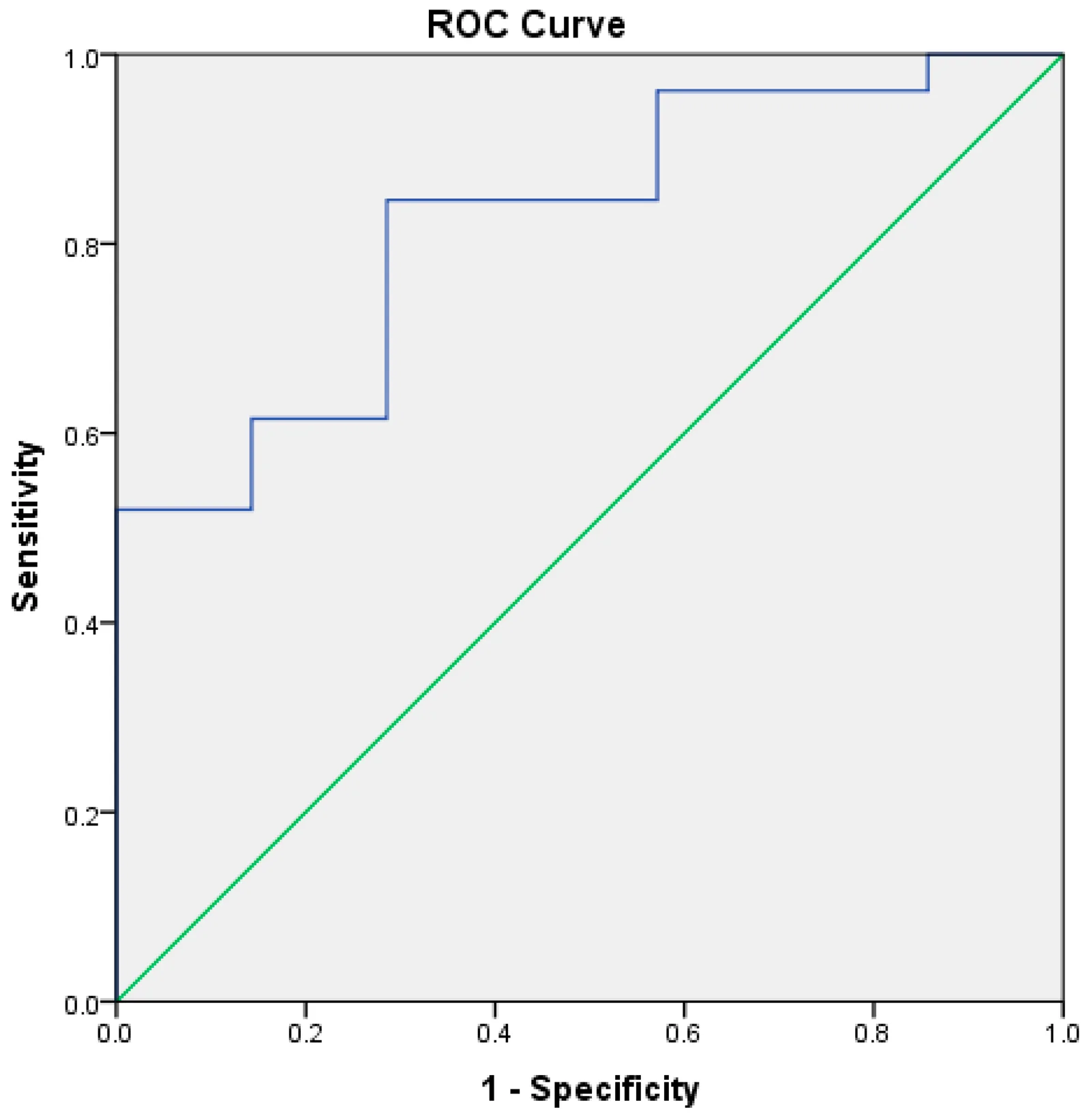
Display of the combined model ROC curve of erythrocytes, hemoglobin, hematocrit, and iron.

The area under the curve, 95% confidence interval, standard error, and *p* value for the iron biomarker in distinguishing patients with RRMS from healthy controls were determined (AUC = 0.728, 95% CI = 0.541–0.916, SE = 0.096, *p* = 0.037). Iron showed acceptable diagnostic potential as a biomarker in distinguishing patients with RRMS from healthy controls. (AUC = 0.728). We presented the combined model of erythrocytes, hemoglobin, hematocrit, and iron in the ROC curve in Figure 1.

**Figure 2:**
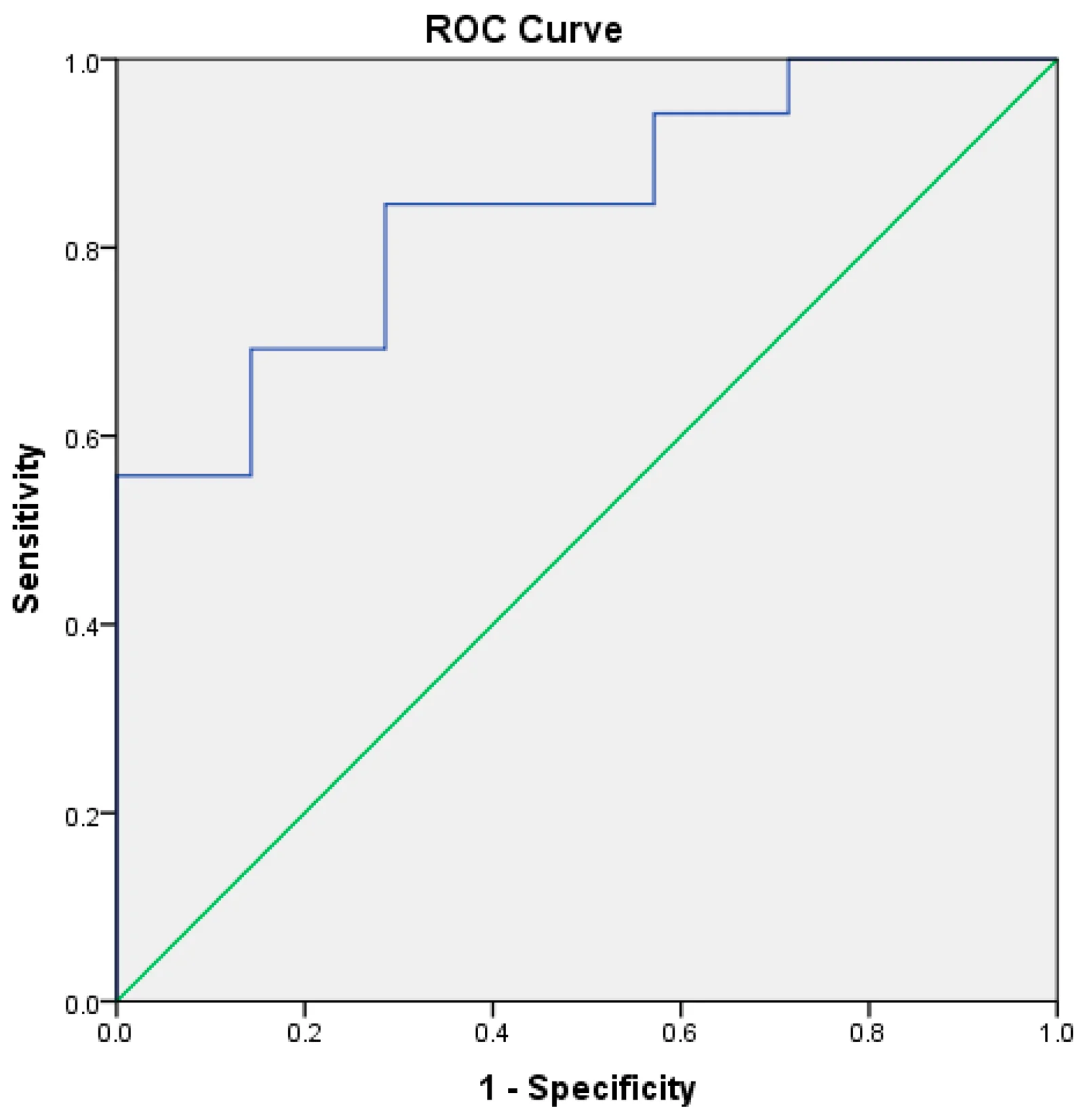
Display of the combined model ROC curve of erythrocytes, hemoglobin, hematocrit, iron, nitric oxide, reduced glutathione, and vitamin D.

The combined model of erythrocytes, hemoglobin, hematocrit, and iron showed excellent diagnostic potential in distinguishing patients with RRMS from healthy ones and is statistically significant because *p* < 0.05 (AUC = 0.821, Std. Error = 0.074, 95% CI = 0.677–0.966, *p* = 0.006). The second combined model of erythrocytes, hemoglobin, hematocrit, iron, nitric oxide, reduced glutathione, and vitamin D is presented in Figure 2.

**Figure 3:**
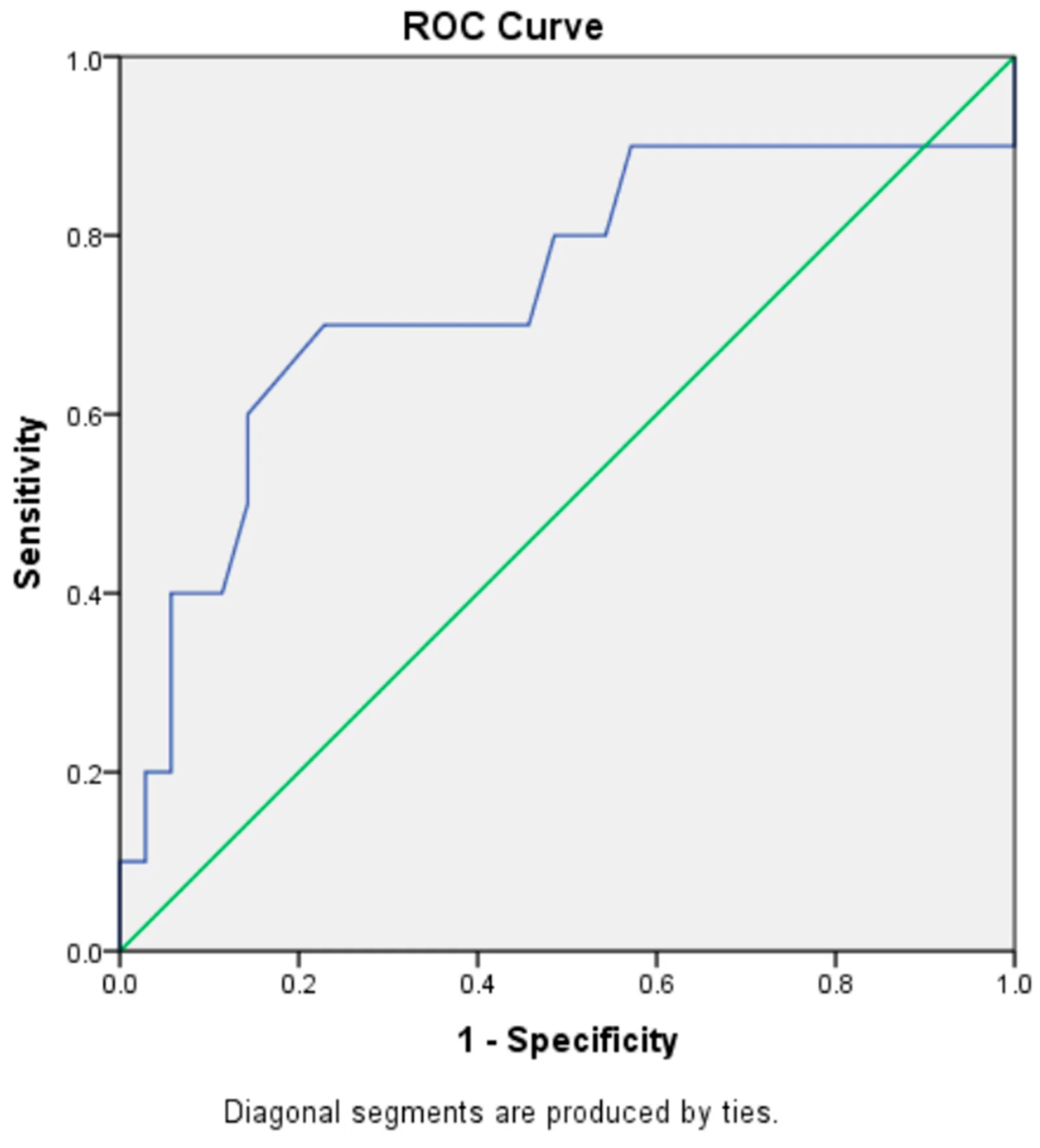
ROC curve representation of iron in distinguishing patients with active MS.

The combined model of erythrocytes, hemoglobin, hematocrit, iron, nitric oxide (indirectly nitrite), reduced glutathione, and vitamin D showed excellent diagnostic potential in distinguishing patients with relapsing-remitting multiple sclerosis from the healthy controls and was statistically significant because *p* < 0.05 (AUC = 0.841, Std. Error = 0.067, 95% CI = 0.710–0.972, *p* = 0.004). The third ROC curve shows the potential of iron in separating active from inactive MS, as shown in Figure 3.

The area under the curve, 95% confidence interval, standard error, and *p* value were determined for the iron biomarker in distinguishing patients with active MS (AUC = 0.737, 95% CI = 0.536–0.939, SE = 0.103, *p* = 0.023). Iron showed acceptable diagnostic potential as a biomarker in distinguishing patients with inactive MS from patients with active MS (AUC = 0.737).

### 3.5. Correlation of Iron with Biochemical Parameters

Serum iron showed a moderate positive correlation with hemoglobin (ρ= 0.453, *p* < 0.001), hematocrit (ρ= 0.405, *p* = 0.001), total bilirubin (ρ= 0.322, *p* = 0.009), and direct bilirubin (ρ= 0.249, *p* = 0.047), and a weak positive correlation with uric acid (ρ=0.267, *p* = 0.038), while a moderate negative correlation was observed with erythrocyte sedimentation rate (ρ=−0.332, *p* = 0.023).

Table 6 presents the results of the univariate and multivariate linear regression analysis of predictors of serum iron levels. Univariate linear regression analyses were performed for all examined variables in order to assess their individual associations with iron levels.

In univariate linear regression analysis, hemoglobin (*p* = 0.001), hematocrit (*p* = 0.003), total bilirubin (*p* = 0.025), and presence of disease (*p* = 0.048) emerged as statistically significant predictors of iron levels, while H_2_O_2_ (*p* = 0.096) and uric acid (*p* = 0.053) showed borderline or non-significant effects.

All predictors that were significant or close to the significance threshold in the univariate analysis were included in the multivariate model. A multivariate linear regression analysis including hemoglobin, hematocrit, total bilirubin, uric acid, H_2_O_2_, and presence of disease demonstrated a statistically significant overall model (F = 4.840, *p* = 0.001), explaining 39.2% of the variance in iron levels (R^2^ = 0.392; adjusted R^2^ = 0.311).

Within this model, the presence of disease (*p* = 0.049), hemoglobin (*p* = 0.038), and total bilirubin (*p* = 0.002) remained statistically significant independent predictors of iron levels (*p* < 0.05), whereas hematocrit (*p* = 0.146), uric acid (*p* = 0.454), and H_2_O_2_ (*p* = 0.115) did not retain statistical significance in the presence of the other variables.

The model also indicated pronounced multicollinearity between hemoglobin and hematocrit (VIF > 10), suggesting substantial overlap in the information provided by these variables.

## 4. Discussion

This study focused on the effect of serum iron levels on various clinical parameters and radiological disease activity in patients with RRMS multiple sclerosis. In our study, a statistically significant difference in serum iron concentration between patients with RRMS and healthy controls was demonstrated. Taking into account the fact that the group of patients with RRMS had a higher average age compared to the controls, we can conclude that a positive correlation of age with iron levels was confirmed. Several previous studies have examined differences in serum iron levels between patients with multiple sclerosis and healthy people. The results of most studies have shown that serum/plasma iron levels were similar in MS patients and controls without a statistically significant difference [53,54,55,56]. In Italy, Visconti et al. examined the relationship between serum iron levels in patients after the first attack of multiple sclerosis and during six months of follow-up compared to healthy controls, and no statistically significant difference was observed [55]. However, the results of a small number of studies have shown that there is a statistically significant difference in the level of serum iron in patients with multiple sclerosis compared to the control group. Contrary to our results, where iron was elevated in patients with RRMS compared to healthy controls, in some studies, serum iron was lower in patients with RRMS compared to healthy controls [16,17,57,58]. Comparison of serum iron concentrations by sex showed higher values in men, close to statistical significance, which is consistent with existing literature [59]. Differences in parameters between the sexes can be explained by the expression of genes encoded by sex chromosomes, the influence of sex hormones on the immune system because there are differences in both innate and acquired immunity, and the hypothalamic-pituitary-adrenal axis. It is known that disease progression occurs more rapidly in men [60]. Sex differences in the age of onset of the disease have been observed, with a female predominance from puberty onwards. It is known that men have a more severe clinical course with a greater degree of disability, motor symptoms and incomplete recovery more often than women. More recent studies have shown a greater degree of atrophy and neurodegenerative changes in men [61]. One study showed a higher degree of disability in men, while in our study no difference in the degree of disability between the sexes was observed. Anxiety was higher in women in that study, which is consistent with our results [62] A study that followed patients with RRMS for 4 years found no sex differences in levels of depression, while our study observed significantly higher levels of depression in women [63].

The results of this study showed that serum iron levels in patients with multiple sclerosis are positively correlated with hemoglobin and hematocrit levels, total bilirubin, direct bilirubin and uric acid, and negatively correlated with sedimentation rate, while they did not correlate with other parameters, such as leukocyte count, erythrocyte count, fibrinogen, UIBC, TIBC, ferritin, CRP, vitamin D, and sedimentation rate. Hemoglobin and total bilirubin were recorded as significant independent predictors. Iron is incorporated into heme, which is a component of hemoglobin. Macrophages phagocytize damaged erythrocytes from the circulation and convert hemoglobin to bilirubin, and iron returns to the circulation, where it binds to transferrin again, which explains the positive correlations of iron with hemoglobin and bilirubin in our study [64]. In the literature, ferritin has shown elevated values in one study from Brazil [65], while in other studies from Iran, Serbia and Austria there was no statistically significant difference [16,52,66], and decreased values in one study from South Africa [67]. In our study, no statistically significant correlation was observed with ferritin, which represents a static iron depot, but is better explained by dynamic parameters such as hemoglobin, hematocrit, bilirubin, uric acid, and sedimentation. Hemoglobin was within normal limits in all patients in some studies [57], while in other studies, decreased values were shown in MS patients [67]. Erythrocyte sedimentation rate (ESR) was within normal values in some studies [68,69], while in other studies it was elevated [70] or reduced [71]. In Turkey, Doğan et al. measured serum iron, ferritin, UIBC, CRP, leukocytes, and sedimentation rate in a study investigating the association with oxidative stress in RRMS. Elevated CRP and ferritin and decreased iron were reported in RRMS patients compared to controls, while leukocytes and sedimentation rate did not show a correlation with iron metabolism parameters [72]. Low-grade inflammation in MS may not be adequately reflected by conventional systemic markers such as ESR and CRP, which are often within normal ranges despite active CNS pathology [73]. The association of serum iron concentration with uric acid has not been directly investigated so far. Uric acid was examined in serum in patients with RRMS and healthy controls, where a significantly higher value was shown in the relapse phase [74]. In another study, serum uric acid was examined according to duration, disability, MRI activity, and gender, but no significant correlation was observed. Uric acid was reduced in all patients with MS compared to controls with other inflammatory and non-inflammatory diseases, and in another study determining risk of cardiovascular events in MS [68,75]. Moccia et al. observed a progressive decrease in uric acid levels over a 2-year period in patients with RRMS [76]. Anđelić et al. reported decreased uric acid and total bilirubin in MS patients compared to healthy controls, and this decrease was more pronounced in women. Uric acid showed a negative correlation with disease duration [77]. In the study by Obradović et al., increased values of malondialdehyde, nitrite, superoxide dismutase, catalase, and decreased uric acid and bilirubin were observed compared to controls [78]. In our study, a positive correlation of uric acid and total and direct bilirubin with iron was obtained, while oxidative parameters did not show a significant correlation. Both bilirubin and uric acid are recognized endogenous antioxidants, and their reduction in MS has been linked to increased oxidative stress [79,80].

The age of the subjects was grouped into three categories (≤30, 31–60, and ≥61 years), and similar age divisions have been used in previous studies. Koch et al. reported a decrease in focal inflammatory activity with age across the entire spectrum of MS [80]. In the study by Stojković et al., iron showed a positive correlation with age [66].

Disease duration was categorized into three groups (0–10, 11–20, and 21–40 years) reflecting early, intermediate, and late disease stages in line with previous models of disease progression [26,27]. Consistent with our findings, the relationship between iron metabolism parameters and the duration of the disease and EDSS score was not confirmed in the study by Knyszyńska et al. [16]. Although the present study did not demonstrate a significant correlation between serum iron levels and the degree of disability, some studies reported the influence of iron loss in the brain tissue and spatial redistribution of iron in the form of iron rim lesions on disability progression [81,82,83].

One of the aims of our study was to investigate whether serum iron levels are associated with radiological disease activity. In our study, a statistically significant difference was observed between the group of patients without new lesions and the group of patients with up to three new lesions, while in the group with more than 3 lesions, there were not enough subjects to calculate medians, so this group of subjects could not be compared with the other two groups. To our knowledge, patients have not previously been stratified according to MRI activity in this manner. The ROC curves showed the diagnostic potential of iron in differentiating active from inactive MS. Previous literature has discussed the role of iron in oxidative stress and mitoc,hondrial dysfunction, which underlie neuroinflammation and neurodegeneration in MS [8,64,84]. Results from several studies suggest a link between iron deposition in brain tissue, primarily in the gray matter, and disease progression, while reduced iron has been reported in normal-appearing white matter and the thalamus [11,19,85,86,87]. In one study, iron levels were reduced in inactive lesions in MS, while in cerebrospinal fluid (CSF), they were similar in MS patients and controls [88]. Our study did not measure iron levels in either lesions or CSF, and this should be considered in future studies. Most neuroradiological studies have examined paramagnetic ring lesions and their association with neurodegeneration, disability progression, and atrophy of brain structures, with some studies including histopathological confirmation of iron deposition in brain regions [12,89,90,91,92]. Reeves et al. investigated the patterns of iron accumulation in patients with MS and concluded that brain atrophy is accelerated in MS, while iron homeostasis is disrupted with a decrease in total brain iron content in MS [91]. Previous studies have shown that iron chelators such as deferoxamine and deferiprone help mobilize iron deposited in lesions and thus reduce its accumulation on MRI scans. We discussed this in a previous literature review [6].

Previous studies reported region and disease-stage-specific associations with age, disease duration, and disability severity. For example, iron levels in the normal-appearing white matter and periventricular lesions have been shown to negatively correlate with age, duration of the disease, and EDSS score, particularly in progressive forms of MS [87,93]. In the present study, no statistically significant correlation of serum iron levels with EDSS score, age groups, and duration of the disease was found. Recent research has shown that disruption of iron homeostasis in MS is primarily related to altered regional distribution rather than to changes in total brain iron content. Although in most studies total brain iron levels reported unchanged or even reduced values in MS, iron redistribution has been proposed as a potential biomarker of disease progression, particularly in lesions and deep gray matter [11,87].

Experimental studies with the EAE model suggest that iron accumulation is involved in ferroptosis-related neurodegeneration. Louqian et al. reported increased iron levels in the spinal cord and cerebral cortex of animals, while pharmacological inhibition of ferroptosis improved clinical outcomes and reduced iron-related oxidative damage. Iron accumulation was not observed in the early disease, indicating that iron may not act as an initial trigger of ferroptosis, but it exacerbates disease progression [93].

Fatigue is a subjective feeling of physical or mental exhaustion that limits activities of daily living and is thought to arise from a combination of neuroinflammation and neurodegeneration [94]. In the present study, serum iron levels showed a negative but non-significant correlation with fatigue scores, and no statistically significant differences were observed when patients were stratified according to fatigue severity. Previous studies have not demonstrated a direct association between iron status and fatigue in MS, and similar negative findings without statistical significance regarding plasma iron concentrations have been reported [95]. Although iron deficiency without anemia has been associated with fatigue in other clinical populations and iron supplementation may reduce fatigue in such conditions, this mechanism does not appear to play a major role in MS-related fatigue [96]. Taken together, these findings suggest that fatigue in MS is likely driven by multifactorial central mechanisms rather than by systemic iron metabolism alone.

Depression is a common comorbidity in MS that contributes to a poorer prognosis [97]. In a study by Oliveira et al., iron showed an inverse correlation with depression scores, and ferritin was elevated in MS patients due to the presence of chronic inflammation, which is directly related to reduced iron levels in depressed MS patients [98]. For the first time, Knyzinska et al. examined the relationship between iron metabolism parameters and the following clinical characteristics in Poland: fatigue, depression, and quality of life in 90 patients with multiple sclerosis. The results of this study showed that low ferritin levels and low hemoglobin were associated with worsening depression and quality of life in patients with multiple sclerosis, while fatigue was inversely proportional to mood and quality of life. A positive correlation was also found between the intensity of depressive symptoms according to the degree of disability with symptoms of fatigue, indicating that the increase in disability and fatigue was accompanied by an increase in depressive symptoms [16]. Our study also showed a negative correlation between iron levels and depression levels, but it did not reach statistical significance. When patients were stratified according to level of depression, no significant differences in serum iron concentration were observed. Importantly, depression in MS appears to be more strongly associated with disability progression and neurodegenerative changes rather than with systemic iron status [31,72,99,100,101]. In the present study, quality of life and sleep quality in patients with RRMS were not examined. Future studies should also include these segments in the research due to the significant impact that fatigue, anxiety, and depression have on sleep and quality of life. Our study also did not compare depression according to the degree of disability.

Pain is a frequent and clinically important symptom in multiple sclerosis, often presenting with neuropathic or mixed characteristics and significantly affecting quality of life [102]. Recent research has reported a high prevalence of neuropathic pain in MS, frequently associated with disability progression, fatigue, and depressive symptoms [103,104,105,106]. In the present study, serum iron levels were not significantly associated with pain intensity or the presence of neuropathic pain. Our results are in line with existing evidence suggesting that pain in MS is primarily driven by central mechanisms related to neuroinflammation, neurodegeneration, and disease burden rather than by systemic iron metabolism. Several limitations were observed regarding pain assessment, as pain subtypes, localization, and MRI correlates were not analyzed. Additionally, interactions between pain, fatigue, and depression were not examined. Future studies integrating detailed pain phenotyping, neuroimaging data, and biochemical markers are warranted to further elucidate the mechanisms underlying pain in MS.

Previous studies have reported that iron intake, hemoglobin, and serum ferritin were negatively correlated with headache in women. In men, no association was found between headache and iron and ferritin [107,108,109]. In the present study, iron was lower in those with present headache, but with no statistical significance, which is in agreement with previous studies.

Anxiety is more common in multiple sclerosis than in the general population and is more associated with disease activity, depression, fatigue, and progression of disability than with systemic biochemical parameters [110,111]. The association of iron metabolism with anxiety has been studied at the gene level, where serum iron, ferritin, and transferrin have been shown to harm the development of anxiety [112]. We did not obtain a statistically significant correlation of serum iron with anxiety as a continuous variable or after stratification according to anxiety severity. A disadvantage of our study would be that we did not examine the localization of lesions, brain regions associated with anxiety, and the relationship with cognition. Although anatomical iron pathways in anxiety are known from neuroimaging studies, their association with serum iron has not yet been proven [113,114,115].

Regarding regional differences in iron levels, Matar et al. conducted a study in Lebanon in which serum iron and zinc levels were measured in 27 MS patients and 42 healthy controls, but no statistically significant difference was shown between MS patients and controls. Similar to our study, controls were on average younger than MS patients [116]. Another study examined the effect of whole-body cryotherapy on serum iron in women with MS in the Australia and New Zealand region, but this study also did not observe a significant difference between the experimental and control groups in serum iron before and after the intervention [117]. In Saudi Arabia, serum iron and ferritin showed no association with disease severity, nor did they differ between genders. Patients were on average younger and most were women, and more than half of the patients had low serum iron [118]. Abo-Krysha et al. found no difference in iron values in 20 MS patients and 10 controls, but serum transferrin was significantly elevated in MS patients compared to controls, which would indicate increased iron turnover, a proinflammatory and pro-oxidant environment [53]. Two studies in Rome, Italy, showed different results, in one the iron levels were similar to controls, while in the other study the iron levels were reduced [55,57]. In Athens, Greece, one study showed that iron, hemoglobin, and transferrin levels were within reference ranges, while soluble transferrin was significantly elevated in active progressive and relapsing forms, which would explain the increased iron turnover [56]. In Vienna, Austria, Bsteh et al. did not find significant differences in iron metabolism parameters between MS patients and controls, nor among MS subgroups, but a significant correlation of iron, ferritin, transferrin with hepcidin was observed [52]. A study in Serbia that examined oxidative stress parameters associated with ferroptosis found no significant difference in iron metabolism parameters between RRMS and PMS [66].

Main limitation of our study would be relatively small control group- the observed effect sizes and confidence intervals support the robustness of the findings. The unequal ratio of the number of subjects between the groups is a consequence of the limited availability of appropriate healthy controls and the focus of this study on the population of patients with multiple sclerosis. Other limitations would be type of research (a cross-sectional study), regional changes in iron concentration were not measured, lack of pathohistological confirmation of iron deposition at the cellular level, level of iron in the cerebrospinal fluid was not measured, pain was not monitored over time, neither pain intensity or quality and examination of cognition was not included. Future research should also include these aspects in order to gain a more complete picture of the role of iron in the activity and progression of MS. Larger prospective studies are needed that would monitor changes in the quality of pain and more subtle changes between symptoms and iron metabolism.

## 5. Conclusions

Our study observed a significant difference in iron values between patients with RRMS and the control group, which has been examined in previous studies. This is the first study in Serbia to examine the association of serum iron with the level of fatigue, anxiety, depression, neuropathic pain, and radiological activity of the disease on MRI. This study is a descriptive attempt to present the potential role of iron in the radiological activity of the disease. The intention of our study was not to draw a final conclusion that iron is a biomarker for sure, but to show that there is a possibility that in further studies with a larger number of patients it could potentially predict radiological activity of the disease, if in this study on a limited number of subjects it showed a significant result. One of the goals of this study was to show how the pathological process unfolds without the influence of drugs.

## Figures and Tables

**Table 1:** Demographic and clinical characteristics of subjects.

Parameter	Relapsing-Remitting Multiple Sclerosis	Control Group
Number of respondents	65	11
Age	40.65 ± 11.66	34 ± 10.90
Weight	72.00 (62.00–82.00)	76.00 (65.25–92.50)
Height	1.74 ± 0.10	1.74 ± 0.09
Body mass index	24.19 ± 4.05	26.83 ± 7.65
Duration of disease	8.80 ± 8.01	/
Age of onset	31.85 ± 8.83	/
Age of diagnosis	6.58 ± 7.23	/
Delay in diagnosis	2.22 ± 3.11	/
Number of relapses per year	0.83 ± 0.55	/
Number of relapses in two years	1.18 ± 0.73	/
EDSS	2.74 ± 1.74	/
PD-Q	2.00 (0.00–10.00)	3.00 (0.00–9.00)
MFIS	31.00 (13.00–49.25)	22.00 (0.00–32.00)
BDI-II	8.00 (2.00–15.00)	4.00 (0.00–11.00)
HAM-A	8.00 (5.00–20.00)	6.00 (2.00–28.00)
VAS	1.00 (0.00–3.00)	2.00 (0.00–4.00)
Iron	15.50 (12.28–18.58)	10.10 (6.08–15.95)

EDSS—Expanded Disability Status Scale, PD-Q—PainDETECT Questionnaire, MFIS—Modified Fatigue Impact Scale, BDI-II—Beck Depression Inventory, HAM-A—Hamilton Anxiety Scale, VAS—Visual Analogue Scale.

**Table 2:** Comparison of variables between subjects with relapsing-remitting form of the disease and the control group.

Variable	Mean Rank RRMS	Mean Rank Controls	U	Z	*p*
Age	40.48	26.82	229.0	−1.899	0.058
Weight	36.27	41.60	269.0	−0.739	0.460
Height	36.76	38.50	300.0	−0.241	0.810
BMI	36.15	42.35	261.5	−0.858	0.391
PD-Q	37.41	38.00	341.0	−0.086	0.931
MFIS	38.68	27.55	237.0	−1.605	0.108
BDI-II	38.98	29.05	253.5	−1.418	0.156
HAM-A	37.75	36.09	331.0	−0.236	0.814
VAS	36.21	44.86	265.5	−1.299	0.194
Iron	35.33	20.25	126.0	−2.084	**0.037** *

Mann–Whitney U test, * significance level: *p* value < 0.05.

**Table 3:** Correlation of iron with clinical parameters, oxidative stress parameters, and questionnaires for assessing depression, anxiety, fatigue, and pain.

Parameter	Correlation Coefficient	*p* Value
Duration of the disease	−0.149	0.263
Clinical activity of multiple sclerosis	0.074	0.619
Number of relapses per year	0.079	0.587
Number of relapses in two years	0.118	0.423
Radiological disease activity (MRI activity)	0.225	0.137
Isoelectric focusing of cerebrospinal fluid and serum	−0.042	0.772
EDSS	−0.058	0.668
Superoxide anion	−0.012	0.924
Hydrogen peroxide	−0.058	0.644
Nitrogen monoxide (indirectly nitrites)	−0.096	0.443
TBARS	−0.123	0.326
Catalase	−0.105	0.400
Superoxide dismutase	0.129	0.301
Reduced glutathione	−0.072	0.565
PD-Q	−0.062	0.621
MFIS	−0.140	0.269
BDI	−0.087	0.490
HAM-A	−0.730	0.564
VAS	−0.118	0.350

Spearman’s rank correlation coefficient.; EDSS—Expanded Disability Status Scale, TBARS—thiobarbituric acid reactive substances, PD-Q—PainDETECT Questionnaire, MFIS—Modified Fatigue Impact Scale, BDI—Beck Depression Inventory, HAM-A—Hamilton Anxiety Scale, VAS—Visual Analogue Scale.

**Table 4:** Iron levels were examined according to sex, age, disease onset, disease duration, clinical activity, disease activity on MRI, presence of oligoclonal bands, and headache.

Parameter	Groups	Iron	*p* Value
Sex	Male	16.90 (12.50–19.10)	0.059
	Female	14.20 (9.80–17.30)	
Age	≤30 years	14.10 (9.98–23.58)	0.913
	31–60 years	15.00 (11.80–17.30)	
	≥61 years	17.20 (9.65–18.35)	
Age of onset	Before 55 years	15.00 (11.87–17.30)	0.629
	After 55 years	17.25 (9.17–19.02)	
Duration of disease	0–10 years	15.70 (12.35–19.10)	0.317
	11–20 years	13.00 (10.15–17.12)	
	21–40 years	17.25 (10.72–22.02)	
Clinical activity of multiple sclerosis	Inactive	15.70 (12.25–17.30)	0.771
	Active	15.30 (12.20–21.10)	
Radiological disease activity (MRI activity)	Without new lesions	15.15 (12.37–17.22)	0.027 *
	Up to 3 new lesions	18.75 (15.02–23.25)	
	More than 3 new lesions	n = 1 (descriptive only)	
Oligoclonal bands	Present	15.70 (12.10–18.40)	0.784
	Not present	14.20 (12.40–17.30)	
Headache	Present	12.40 (7.02–15.40)	0.070
	Not present	16.60 (12.20–18.90)	

Comparisons of numerical variables between two independent groups were performed using the Mann–Whitney U test, while comparisons between three independent groups were performed using the Kruskal–Wallis test; * significance level: *p* value < 0.05.

**Table 5:** Post hoc test.

Comparison of Groups According to MR Activity	Mean Rank Group 1	Mean Rank Group 2	U	Z	*p*
No new changes vs. up to 3 new lesions	20.18	30.40	91.0	−2.214	0.027 *
No new changes vs. more than 3 new lesions	18.47	2.00	1.0	−1.586	0.113
Up to 3 new lesions vs. more than 3 new lesions	6.40	2.00	1.0	−1.265	0.206

* Significance level: *p* < 0.05.

**Table 6:** Univariate and multivariate linear regression analysis of predictors of serum iron levels.

Predictor	Univariate Analysis R	R^2^	Adj. R^2^	F	*p*	β/B	Multivariate Analysis B	SE	β	t	*p*	Tolerance	VIF
H2O2	0.208	0.043	0.028	2.855	0.096	−0.208	3.718	2.311	0.391	1.609	0.115	0.229	4.365
Hemoglobin	0.410	0.168	0.154	11.518	0.001 *	0.410	0.500	0.234	1.125	2.135	0.038 *	0.049	20.556
Hematocrit	0.381	0.145	0.130	9.693	0.003 *	0.381	−127.200	85.965	−0.785	−1.480	0.146	0.048	20.840
Total bilirubin	0.281	0.079	0.064	5.307	0.025 *	0.281	0.647	0.199	0.408	3.254	0.002 *	0.858	1.165
Uric acid	0.249	0.062	0.046	3.893	0.053	0.249	−0.007	0.010	−0.097	−0.756	0.454 *	0.812	1.231
Presence of disease	0.244	0.060	0.045	4.052	0.048 *	−0.244	−4.424	2.186	−0.490	−2.023	0.049 *	0.231	4.338

* Significance level: *p* < 0.05.

## Data Availability

Data is contained within the article.

## Abbreviations

The following abbreviations are used in this manuscript:

BDI	Beck Depression Inventory
HAM-A	Hamilton Anxiety Rating Scale
MFIS	Modified Fatigue Impact Scale
PD-Q	PainDETECT Questionnaire
VAS	Visual Analogue Scale
CNS	central nervous system
RRMS	relapsing-remitting multiple sclerosis
RAW	relapse-associated worsening
PIRA	progression independent of relapse activity
SPMS	secondary progressive multiple sclerosis
PPMS	primary progressive multiple sclerosis
ARR	annual relapse rate
MRI	magnetic resonance imaging
EAE	experimental autoimmune encephalomyelitis
EDSS	Kurtzke’s Extended Disability Status Scale
CRP	C-reactive protein
TBARS	thiobarbituric acid
NO_2_^−^	nitrites
O_2_^−^	superoxide anion radical
H_2_O_2_	hydrogen peroxide
CAT	catalase
SOD	superoxide dismutase
GSH	reduced glutathione
DSM-IV	Diagnostic and Statistical Manual of Mental Disorders,4-th edition
MSQLI	Multiple Sclerosis Quality of Life Inventory
FIS	Fatigue Impact Scale
AUC	area under the curve
CI	confidentiality interval
SE	standardized error
ROC	receiver operating characteristic
UIBC	unsaturated iron binding capacity
TIBC	total iron binding capacity
ESR	erythrocyte sedimentation rate
CSF	cerebrospinal fluid
m	male
f	female
